# Repurposing of drugs for triple negative breast cancer: an overview

**DOI:** 10.3332/ecancer.2020.1071

**Published:** 2020-07-13

**Authors:** Andrea Spini, Sandra Donnini, Pan Pantziarka, Sergio Crispino, Marina Ziche

**Affiliations:** 1 Department of Medicine, Surgery and Neuroscience, University of Siena, Siena 53100, Italy; 2 Service de Pharmacologie Médicale, INSERM U1219, University of Bordeaux, Bordeaux 33000, France; 3 Department of Life Sciences, University of Siena, Siena 53100, Italy; 4 Anticancer Fund, Strombeek Bever 1853, Belgium; 5 ASSO, Siena, Italy

**Keywords:** triple negative breast cancer, repositioning, non-cancer drug, preclinical studies, clinical studies

## Abstract

Breast cancer (BC) is the most frequent cancer among women in the world and it remains a leading cause of cancer death in women globally. Among BCs, triple negative breast cancer (TNBC) is the most aggressive, and for its histochemical and molecular characteristics is also the one whose therapeutic opportunities are most limited. The REpurposing Drugs in Oncology (ReDO) project investigates the potential use of off patent non-cancer drugs as sources of new cancer therapies. Repurposing of old non-cancer drugs, clinically approved, off patent and with known targets into oncological indications, offers potentially cheaper effective and safe drugs. In line with this project, this article describes a comprehensive overview of preclinical or clinical evidence of drugs included in the ReDO database and/or PubMed for repurposing as anticancer drugs into TNBC therapeutic treatments.

## Background

Breast cancer (BC) is the most frequent cancer among women in the world. Triple negative breast cancer (TNBC) is a type of BC that does not express oestrogen receptors, progesterone receptors and epidermal growth factor receptors-2/Neu (HER2) and accounts for the 16% of BCs approximatively [1, 2]. Due to its lack of response to hormone and targeted therapies, the number of therapeutic opportunities is limited [3, 4]. TNBC patients are difficult to treat, with unfavourable prognosis and are generally administered with the standard chemotherapy. At the moment, novel treatment approaches, such as immunotherapy, as well the repurposing of old drugs currently used for indications other than TNBC, is under investigation. In this context, we have previously reviewed the preclinical and clinical anticancer efficacy and safety of beta blockers in TNBC [5].

Drug repurposing is the application of an old drug to a new disease indication: this holds the promise of rapid clinical impact at a lower cost than de novo drug development [6]. In oncology, where new treatments in the last years are becoming more expensive due to the introduction of innovative therapies such as targeted therapies and immunotherapies, there is an increased interest at the use of already clinically approved non-cancer drugs, off patent and with known targets, as possible cancer treatments [7]. One study published by Pantziarka *et al* [8], point the spotlight on this matter building up a project about drug repurposing in the treatment of cancer. The REpurposing Drugs in Oncology (ReDO) project investigates the potential use of licensed non-cancer medications as sources of new cancer strategies. ReDO project has used a literature-based approach to identify licensed non-cancer drugs with published evidence of anticancer activity. At present, data of 268 drugs have been included in the REDO database (ReDO_DB) [8].

In line with this project, we searched in PubMed for published preclinical or clinical evidence of anticancer activity for all drugs included in the ReDO_DB for TNBC. Specifically, starting from each drug present in ReDO_DB, we searched in PubMed for published preclinical and clinical evidence of anticancer activity for TNBC. The strings were composed by the name of the drugs and specific keywords related to TNBC.

An additional search string was used to investigate potential clinical evidence about drugs not included in ReDO_DB or references not retrieved in the first search. The string was composed by three blocks concerning keywords related to TNBC, repurposing and study type, respectively. Both strings are provided in the supplementary file (Table S1). Observational or clinical trials for which a TNBC cohort was defined were included. The articles that were not written in English were excluded.

Moreover, clinicaltrials.gov [9] was searched for ongoing or completed clinical studies on drug repurposing and TNBC. All searches were performed on March 2019, and the information extracted were the following: 1) preclinical studies: number of studies per drug and pharmacological activity; 2) clinical studies: study type, country, study period, population studies, exclusion criteria, age, follow up, arms, treatments and outcomes; 3) clinicaltrials.gov: number of studies per drug.

The aim of this paper is to give to clinicians and scientists a comprehensive overview about preclinical and clinical studies, including clinical trials, present in literature on the repurposing of old-licensed drugs for TNBC.

We found 188 preclinical studies references (see Supplementary Material), 18 clinical references [10–26] and 16 references on clinical trials.gov on drug repurposing for TNBC [9].

### Preclinical studies

Using the PubMed database, we found preclinical evidence on TNBC models (cell lines and xenograft models of TNBC) for 84 out of 268 old drugs (31.3%) present in the ReDO_DB. For 42 of the 84 drugs, only one reference was retrieved (Table S2). Thirteen studies referred to the anti-proliferative, pro-apoptotic and immune-stimulating effects of metformin, thirteen to the cytotoxic and anti-metastatic effects of chloroquine, eleven to the anti-proliferative and anti-invasive effects of simvastatin, eight to the anti-inflammatory and anti-angiogenic effects of acid acetylsalicylic and eight studies to the anti-angiogenic, anti-proliferative and anti-apoptotic effects of zoledronic acid. Main indications for drugs with preclinical evidence of efficacy on TNBC model were various and heterogeneous including epilepsy, analgesia, hypertension, diabetes, insomnia and other.

### Clinical studies

**Figure 1.**
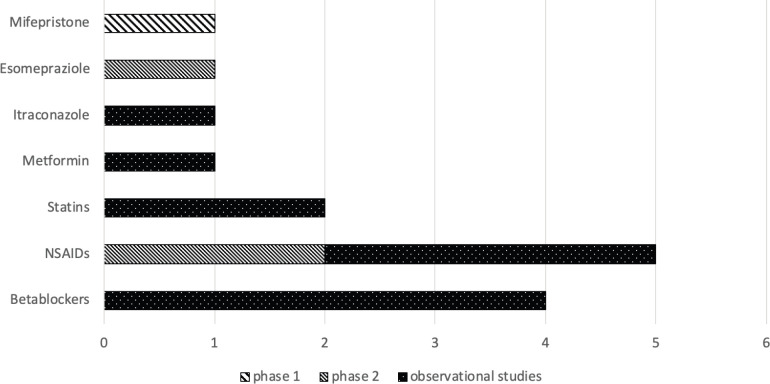
Type of studies per drug. This shows the number of clinical trials (only phase 1 and 2 studies were found) and observational studies conducted per drug/pharmacological classes.

Table 1 shows all 17 clinical references collected (the article of Spera *et al* analyses two different retrospective studies on beta blockers efficacy and safety on TNBC [13], and the articles of Hagasewa *et al* [15] and Ishikawa *et al* [16] analysed the same cohort of patients). Clinical evidence on twelve licensed drugs was found, and of these drugs, eleven out of 268 (4.1%) were included in ReDO_DB. Eleven studies out of 18 were retrospective studies [10–13, 17, 19, 20, 22, 25, 26], six were phase II and [14–16, 18, 21, 23] one was a phase I clinical trial [24] (see Figure 1 for more details). Retrospective studies ranged from 1995 to 2016, and six out of eleven studies analysed a USA cohort of patients [10, 12, 19, 20, 25, 26]. Eight studies were performed using medical records [10, 12, 17, 19, 20, 22, 25, 26], one was based on disease registries [11] and two reported the results of previous clinical trials [13]. Of the 18 clinical studies collected, four analysed the efficacy of beta blockers (BB) [11–13], five of non-steroidal anti-inflammatory drugs (NSAIDs) [17–21], two of zoledronic acid [15, 16], one of metformin [10], one of tetramolybdate [14], one of itraconazole [22], one of esomeprazole [23], one of mifepristone [24] and two of statins [25, 26]. Outcomes retrieved from clinical studies were grouped, whenever possible, in pharmacological categories and summarised in Table 2.

#### Beta blockers (BBs)

BBs were evaluated on postmenopausal women with operated early primary TNBC, on women with invasive TNBC (receiving neoadjuvant chemotherapy), and on women with advanced or nodal positive TNBC. Study populations ranged from 35 patients to 1,417 patients. In the study of Melhem-Bertrandt *et al* [12], using medical chart and pharmacy data from the Breast Cancer Management System Database in the USA, women with invasive TNBC receiving neoadjuvant chemotherapy plus BBs were compared to patients receiving only neoadjuvant chemotherapy between 1995 and 2007. Hazard ratio of recurrence free survival for women administered with chemotherapy plus BBs was 0.30 (95% CI, 0.10–0.87; *p* = 0.027) and hazard ratio of overall survival was 0.35 (95% CI, 0.12–1.00; *p* = 0.05) [12]. Also, in the retrospective study of Botteri *et al* [11] using Breast Cancer and Cardiology Division Databases in Italy and analysing 800 postmenopausal women diagnosed and operated for early primary TNBC between 1997 and 2008, BB users showed significant benefit when compared to not BB users. Breast cancer related events where lower in BB users (13.6% versus 27.9%; *p* = 0.02) and hazard ratio of metastasis and BC death were significant (0.32: 95% CI 0.12–0.90; *p* = 0.031; 0.42: 95% CI 0.18–0.97; *p* = 0.042, respectively). The study of Spera *et al* [13], using data of a randomised, double blind clinical trial (ROSE/TRIO-012), showed significant benefit in women with advanced TNBC using BBs when compared to not users about progression free survival (Hazard ratio = 0.52; 95% CI, 0.34–0.80; *p* = 0.002) but not in overall survival (Hazard ratio = 0.87; 95% CI 0.58–1.31; *p* = 0.504). The second study presented by Spera *et al* [13] using also data from another randomised, double blind clinical trial (BCIRG-005) about women with node positive TNBC did not show any significant benefit of relapse free survival and overall survival (Hazard ratio = 0.69; 95% CI, 0.35–1.34; *p* = 0.269; 0.73; 95% CI, 0.35–1.48; *p* = 0.38, respectively).

#### Metformin

The retrospective study of Bayraktar *et al* [10] using medical chart and pharmacy data from the Breast Cancer Management System Database compared women who received adjuvant chemotherapy with or without metformin in the USA between 1995 and 2007. In total, 1,448 patients (63 diabetic patients receiving metformin, 67 diabetic patients not receiving metformin and 1318 not diabetic patients). The 5 years survival estimates for distant metastasis free survival were 73% in the metformin group, 66% in the non-metformin group and 60% in the non-diabetic group (*p* = 0.23). Overall survival was 67% in the metformin group, 69% in the non-metformin group and 66% in the non-diabetic group (*p* = 0.58). Recurrence free survival was 65% in the metformin group, 64% in the non-metformin group and 54% in the non-diabetic group (0.38). Also, after adjustments, no significant survival outcomes were obtained.

#### Tetramolybdate

The primary endpoint of phase II open label single arm study of Chan *et al* [14] was to assess the change in VEGFR2+ endothelial progenitor cells in women treated with tetrathiomolybdate. The study, performed on 36 women with stage II/III TNBC during adjuvant setting, showed that two year event free survival was 90%.

#### Zoledronic acid

The articles of Hasegawa *et al* [15] and Ishikawa [16] referred to the same phase II, open label, randomised study but analysed different outcomes in the same cohort of patients (34 women with stage IIA/IIIB TNBC) treated with zoledronic acid plus chemotherapy versus chemotherapy in neoadjuvant setting. Pathological complete response was not significant (*p* = 0.112) when comparing neoadjuvant chemotherapy plus zoledronic acid (6/17 (35.3%) CI: 12.6–58.0) with chemotherapy alone (2/17 (11.8%) CI: 0.0–27.1). Also for the 3 years disease free survival, neoadjuvant chemotherapy plus zoledronic acid showed no significant benefit compared to the neoadjuvant treatment alone (*p* = 0.077) despite the fact that the percentage of patients in treatment with zoledronic acid was higher compared to the other arm (94.1% versus 70.6%).

#### NSAIDs

Celecoxib was analysed in two studies: the first, a phase II randomised study of Pierga *et al* [21] performed between 2004 and 2007, analysed 23 women with stage II/III TNBC comparing chemotherapy alone with chemotherapy plus celecoxib. The authors stated that celecoxib did not improve pathological complete response rates, but no specific comparison on this outcome were shown in the article for TNBC patients. The second study, a phase II multicentre open-label single arm study of Chow *et al* [18], analysed women with primary breast cancer. Unfortunately, only two patients with primary TNBC were included and authors could not show any result about this cohort.

Aspirin was analysed in two retrospective studies. The first retrospective study of Shiao *et al* [19] that collected medical records from University of Texas Southwestern TNBC registry, analysed a cohort of 222 women with stage II/III TNBC in the USA between 2005 abd 2013. Sixty-five women were treated with anti-platelet therapy (as aspirin or clopidogrel) and 157 with no anti-platelet therapy. A percentage of patients in both arms (6.3% and 7.1%, respectively) did not receive chemotherapy. Five years disease free survival and 5 years distant metastasis hazard ratios was significantly improved in favour of the first arm (anti-platelet 80.4%, no anti-platelet 62.3%, HR: 0.503 (0.261–0.970); *p* = 0.04; anti-platelet 8.8%, no anti-platelet 31.9%, HR: 0.310 (0.132–0.729); *p* = 0.007, respectively). Five years overall survival hazard ratio was not significant between the two arms (HR: 0.652 (0.343–1.239); *p* = 0.192). The second retrospective study of Williams *et al* [20] performed in USA used electronic medical records of 147 women with primary operable stages I-III TNBC (114 never used aspirin, 19 before diagnosis, and 14 after diagnosis) to analyse overall survival and disease-free survival between 2005 and 2013. Results of this study indicated that aspirin may have an impact on the pathogenesis of TNBC but do not seem to affect breast cancer survival when used after cancer diagnosis (results were presented only for the total cohort of breast cancer patients and not for TNBC subtype).

Finally, Retsky *et al* [17] showed the updated results of a retrospective study performed in Belgium using medical records between 2003 and 2008 [27], in which ketorolac plus chemotherapy was compared to chemotherapy alone in women who underwent mastectomy with axillary dissection. No information about the cohort (as for the number of patients with TNBC, age, etc…) was reported. Also, for the results the authors said that the group receiving chemotherapy plus ketorolac showed a ‘far superior disease free survival in the first few years after surgery’ but no data were shown in particular about TNBC.

#### Itraconazole

The article of Tsubamoto *et al* [22] reported the results of a retrospective study that used medical records of the Kohan hospital in Japan between 2008 and 2012 to analye response rate, median progression-free survival and median overall survival of thirteen patients. TNBC patients who progressed after prior chemotherapy were treated with chemotherapy in combination with itraconazole. No comparison was made. The authors showed that response rate was 62% ([CI], 35%–88%), progression free survival was 10.8 months (95%CI, 7.6–15.3) and overall survival was 20.4 months (95%CI: 13.1–41.4 months).

#### Esomeprazole

The phase II, open label, randomised study of Wang *et al* [23] analysed a cohort of 15 women with metastatic or recurrent TNBC (seven receiving only chemotherapy, two esomeprazole low dose and six esomeprazole high dose). The authors showed that the time to progression of patients receiving esomeprazole when compared to chemotherapy was significantly higher (10.7 versus 5.8 months; *p* = 0.011).

#### Mifepristone

In the Phase I, randomised study of Nanda and colleagues performed in USA, four women with metastatic or locally advanced TNBC were analysed (those patients were allocated to mifepristone plus paclitaxel or placebo). Unfortunately, no information about patients allocation, nor any outcome information could be retrieved from this article [24].

#### Statins

The retrospective study of Shaitelman *et al* [26] used medical records from the MD Anderson Cancer Centre to investigate if women with stage I–III TNBC receiving statins at any time from diagnosis. The authors showed that patients receiving statins did not get any advantage compared to the non-statin users group (0.82 (0.57–1.16); 0.70 (0.47–1.03) relative risk of recurrence and breast cancer death, respectively); when a multivariate analysis was performed (taking in consideration cholesterol and triglyceride values, stage and chemotherapy, the authors showed that statin use was predictive for OS (HR: 0.10, *p* = 0.026, 95% CI: 0.01–0.76).

The retrospective study of Lacerda *et al* [25] using Breast Cancer Management database at MD Anderson Cancer Centre in USA between 1995 and 2011, analysed the risk of loco-regional recurrence at 3 years associated to the use of statins, in patients with inflammatory breast cancer who received adjuvant post-mastectomy radiotherapy. 102 patients underwent post-mastectomy radiation (86 patients) or post-mastectomy radiation plus statins (16 patients). Unfortunately no information about the outcome in TNBC patients was shown.

### Clinicaltrials.gov

Searching the web site of clinicaltrials.gov (clinicaltrials.gov), we found only 17 drugs out of 286 presented in the ReDo_DB with ongoing or completed clinical trials for TNBC. Table 3 shows the list of trials and the recruitment status for each drug. As shown in Table 3, most part of the drugs present only one or few studies published on this website. In total, three studies are recruiting for the assessment of atorvastatin, two for metformin, two for mifepristone, and three for zoledronic acid.

## Future directions

This review presents an overview of all the evidences about the repurposing of old, licensed, non-cancer-drugs in the treatment of TNBC, starting from preclinical evidence and going through current clinical trials. ReDO is an ambitious project aiming to investigate the repurposing of non-cancer-drugs in oncology, and ReDO_DB is a powerful tool that need to be dynamically implemented with recent findings, by adding to the database new drugs for which there are preclinical evidence, and by giving visitors a specific PubMed search string for each tumour and tumour subtypes. The ReDO approach is based on published literature and does not aim to identify new active compounds against cancer. Thus, the database does not include potential repurposing candidates identified through *in silico* modelling or other computational pharmacological approaches that, despite the interest for the research [28–31], unless validated by preclinical studies, represent only future hypothetical repurposed drugs and far from the aim of the ReDO project. The project, in particular, aims to drive scientist attention to investigate already approved non cancer-drugs in the oncology setting. Using this ReDO_DB, we found out that despite a lot of preclinical evidence was produced for drugs included in the database for the treatment of TNBC, only few of them were tested in clinical trials. Moreover, in clinical trials only few of the studies used a large sample of cases and gave explicit results on the repurposing of old drugs for TNBC. Some of the studies did not report any result for TNBC cohort when this is a part of a bigger BC cohort.

Beta Blockers (BBs) seem to be the more promising drugs in the repurposing for the treatment of TNBC. Three articles showed significant benefits of these drugs in women with advanced TNBC and in early primary TNBC patients treated with the combination of chemotherapy plus BBs [11–13]. Unfortunately, in clinicaltrials.gov we found no studies that specifically attempt to evaluate BBs within clinical trials for TNBC patients. One triple blinded phase II randomised trial evaluated the use of pre-operative propranolol (seven days before surgery) compared to placebo in 60 women with early stage surgically-resectable breast cancer. [32]. The authors showed that the treatment with propranolol reduced intra-tumoral mesenchymal transition and promoted immune cell infiltration reducing biomarkers associated with metastatic potential. Unfortunately, authors did not present results stratified for breast cancer sub-type.

While BBs demonstrated to be beneficial in the treatment of TNBC, metformin, a promising molecule in preclinical studies, did not show any efficacy in the treatment of women with TNBC. Bayraktar *et al* [10] showed that metformin does not improve survival outcomes in a population of TNBC women when compared to not users. Of note, two studies on the use of metformin in clinicaltrials.gov on TNBC patients are ongoing.

The articles of Shiao *et al* [19] and Williams *et al* [20] showed conflicting results on aspirin. While the first study showed a significant survival benefit in women with stage II/III by the use of aspirin, Williams *et al* [20] did not show this benefit in the breast cancer population examined (women with operable stage I-III TNBC).

Despite many studies trying to evaluate the use of statins in breast cancer treatment [33–36], in the literature search on PubMed, we retrieved only two retrospective studies on their use in the TNBC cohort. The article of Shaitelman *et al* [26] reported a non-significant improvement of OS for patients in the statin group (with the exception of the multivariate analysis), while the second study of Lacerda *et al* [25] did not show any results for TNBC patients.

Other authors showed significant results on the survival of TNBC patients treated with esomeprazole. Recently, one phase II study on activity of omeprazole on patients with operable TNBC independent of baseline Fatty acid synthase (FASN) expression was presented at the ASCO meeting. [37] *In vitro*, proton pump inhibitors inhibit FASN activity and induce apoptosis in breast cancer cell lines. In this study, omeprazole in combination with anthracycline-taxane (AC-T) was administered to 42 patients until surgery, and pathologic complete response (pCR) was investigated. FASN positivity significantly decreased with omeprazole from 0.53 (SD = 0.25) at baseline to 0.38 (SD = 0.30; *p* = 0.02), and the drug was well tolerated with no known grade 3 or 4 toxicities. Furthermore, the pCR rate was 71.4% (95% CI: 51.3–86.8) in FASN+ patients and 71.8 % (95% CI: 55.1–85.0) in all enrolled patients, demonstrating that the omeprazole in addition to neoadjuvant AC-T yields a promising pCR rate without adding toxicity.

For those drugs collected in ReDO_DB with favourable preclinical evidence or whose retrospective clinical trials were not so large to provide strong evidence, large retrospective cohort studies are needed to evaluate effectiveness. Further, as for BBs that have proven by retrospective studies to be effective in the treatment of TNBC patients, randomised clinical trials might be important to confirm the evidence of the repurposing.

## Final remarks

Drug repurposing is a highly interesting novel strategy for the oncology community and ReDO_DB is a powerful tool that can give authors the opportunity to investigate weather non-anticancer drugs might be effective in cancer treatment. Some precision medicine studies, based on omics data, have included repurposed drugs and have reported interesting case reports of responses from patients [38, 39], however no one on TNBC. Due to the low number of therapeutic opportunities approved for TNBC, repurposing of old drugs seems a valuable approach for this particular type of cancer.

From the literature retrieved, BBs seemed to be the more promising drugs for the repurposing, while evidence about other drugs as NSAIDs still need to be assessed or proven for the treatment of TNBC.

## Conflicts of interest

The authors declare that they have no conflict of interest

## Authors' contributions

MZ and SC conceived the study. AS extracted the data. SD supervised the data extraction. MZ, SD, SC, AS, and PP contributed to the interpretation and discussion of study results. AS and SD drafted the manuscripts. All authors revised and approved the final version of the paper.

## Funding

This study was supported by Fondazione Decima Regio ‘Olga e Raimondo Curri’, Via Cimarra 44-B, Roma.

## Figures and Tables

**Table S1.** Search strings.

**PubMED String: 3 blocks combined with AND**
Pathology block
"Triple negative breast cancer"[Title/Abstract] OR "TNBC"[Title/Abstract] OR "Triple negative breast neoplasms"[Mesh]
Intervention Block
"Repurposing"[All Fields] OR "Repurpose"[All Fields] OR "Repositioning"[All fields] OR "Reposition"[All Fields]
Type of study Block
"Clinical trial"[Publication type] OR "Clinical Study"[Publication Type] OR "Epidemiologic Studies"[Mesh]

**PubMED sting based on ReDO_DB: 2 blocks combined with AND**
Drugs block: all the drugs and their synonyms in the Redo Database
Pathology block
"Triple negative breast cancer"[Title/Abstract] OR "TNBC"[Title/Abstract] OR "Triple negative breast neoplasms"[Mesh]

**Table S2.** Preclinical references for repurposing of drugs for TNBC by ReDO DB.

Drugs	Main indication	Mechanism of action	References
Acetaminophen	Analgesia	TRPA1 inhibitor	–Afshar E, Hashemi-Arabi M, Salami S, Peirouvi T, Pouriran R. Screening of acetaminophen-induced alterations in epithelial-to-mesenchymal transition-related expression of microRNAs in a model of stem-like triple-negative breast cancer cells: The possible functional impacts. Gene. 2019 Jun 20;702:46–55.
Acetazolamide	Glaucoma, diuretic, epilepsy	Carbonic anhydrase inhibitor	–Ivanova L, Zandberga E, Siliņa K, Kalniņa Z, Ābols A, Endzeliņš E, et al. Prognostic relevance of carbonic anhydrase IX expression is distinct in various subtypes of breast cancer and its silencing suppresses self-renewal capacity of breast cancer cells. Cancer Chemother Pharmacol. 2015 Feb;75(2):235–46 –Tatiparti K, Sau S, Gawde KA, Iyer AK. Copper-Free “Click” Chemistry-Based Synthesis and Characterisation of Carbonic Anhydrase-IX Anchored Albumin-Paclitaxel Nanoparticles for Targeting Tumor Hypoxia. Int J Mol Sci. 2018 Mar 13;19(3).
Acetylsalicylic acid	Analgesia, swelling, prophylaxis of venous embolism and further heart attacks or strokes	Cyclooxygenase inhibitor	–Bhardwaj A, Singh H, Trinidad CM, Albarracin CT, Hunt KK, Bedrosian I. The isomiR-140-3p-regulated mevalonic acid pathway as a potential target for prevention of triple negative breast cancer. Breast Cancer Res. 2018 11;20(1):150. –Amaral MEA, Nery LR, Leite CE, de Azevedo Junior WF, Campos MM. Pre-clinical effects of metformin and aspirin on the cell lines of different breast cancer subtypes. Invest New Drugs. 2018;36(5):782–96. –Talarico G, Orecchioni S, Dallaglio K, Reggiani F, Mancuso P, Calleri A, et al. Aspirin and atenolol enhance metformin activity against breast cancer by targeting both neoplastic and microenvironment cells. Sci Rep. 2016 Jan 5;6:18673. –Maity G, Chakraborty J, Ghosh A, Haque I, Banerjee S, Banerjee SK. Aspirin suppresses tumor cell-induced angiogenesis and their incongruity. J Cell Commun Signal. 2019 Jan 4 –Hsieh C-C, Wang C-H. Aspirin Disrupts the Crosstalk of Angiogenic and Inflammatory Cytokines between 4T1 Breast Cancer Cells and Macrophages. Mediators Inflamm. 2018;2018:6380643 –Basudhar D, Glynn SA, Greer M, Somasundaram V, No JH, Scheiblin DA, et al. Coexpression of NOS2 and COX2 accelerates tumor growth and reduces survival in estrogen receptor-negative breast cancer. Proc Natl Acad Sci USA. 2017 05;114(49):13030–5 –Lee YR, Kim KM, Jeon BH, Choi S. Extracellularly secreted APE1/Ref-1 triggers apoptosis in triple-negative breast cancer cells via RAGE binding, which is mediated through acetylation. Oncotarget. 2015 Sep 15;6(27):23383–98 –Chattopadhyay M, Kodela R, Nath N, Barsegian A, Boring D, Kashfi K. Hydrogen sulfide-releasing aspirin suppresses NF-κB signaling in estrogen receptor negative breast cancer cells in vitro and in vivo. Biochem Pharmacol. 2012 Mar 15;83(6):723–32
Albendazole	Parasitic infection	Tubulin polymerisation inhibitor	–Priotti J, Baglioni MV, García A, Rico MJ, Leonardi D, Lamas MC, et al. Repositioning of Anti-parasitic Drugs in Cyclodextrin Inclusion Complexes for Treatment of Triple-Negative Breast Cancer. AAPS PharmSciTech. 2018 Nov;19(8):3734–41.
Amiloride	In congestive heart failure or hypertension treated with thiazides, to conserve potassium	Sodium channel blocker	–Amith SR, Wilkinson JM, Baksh S, Fliegel L. The Na⁺/H⁺ exchanger (NHE1) as a novel co-adjuvant target in paclitaxel therapy of triple-negative breast cancer cells. Oncotarget. 2015 Jan 20;6(2):1262-75.
Aprepitant	Nausea, vomiting	Tachykinin antagonist	–Robinson P, Kasembeli M, Bharadwaj U, Engineer N, Eckols KT, Tweardy DJ. Substance P Receptor Signaling Mediates Doxorubicin-Induced Cardiomyocyte Apoptosis and Triple-Negative Breast Cancer Chemoresistance. Biomed Res Int. 2016;2016:1959270
Artesunate	Malaria	DNA synthesis inhibitor	–Greenshields AL, Fernando W, Hoskin DW. The anti-malarial drug artesunate causes cell cycle arrest and apoptosis of triple-negative MDA-MB-468 and HER2-enriched SK-BR-3 breast cancer cells. Exp Mol Pathol. 2019;107:10–22.
Ascorbic acid	Scurvy	Antioxidant	–Wu C-W, Liu H-C, Yu Y-L, Hung Y-T, Wei C-W, Yiang G-T. Combined treatment with vitamin C and methotrexate inhibits triple-negative breast cancer cell growth by increasing H2O2 accumulation and activating caspase-3 and p38 pathways. Oncol Rep. 2017 Apr;37(4):2177–84. –Hatem E, Azzi S, El Banna N, He T, Heneman-Masurel A, Vernis L, et al. Auranofin/Vitamin C: A Novel Drug Combination Targeting Triple-Negative Breast Cancer. J Natl Cancer Inst. 2018 Nov 20.
Atenolol	Hypertension, angina pectoris	Adrenergic receptor antagonist	–Talarico G, Orecchioni S, Dallaglio K, Reggiani F, Mancuso P, Calleri A, et al. Aspirin and atenolol enhance metformin activity against breast cancer by targeting both neoplastic and microenvironment cells. Sci Rep. 2016 Jan 5;6:18673
Atorvastatin	Coronary heart disease, acute coronary syndrome	HMGCR inhibitor	–Rachner TD, Göbel A, Thiele S, Rauner M, Benad-Mehner P, Hadji P, et al. Dickkopf-1 is regulated by the mevalonate pathway in breast cancer. Breast Cancer Res. 2014 Feb 14;16(1):R20 –Mafuvadze B, Liang Y, Hyder SM. Cholesterol synthesis inhibitor RO 48-8071 suppresses transcriptional activity of human estrogen and androgen receptor. Oncol Rep. 2014 Oct;32(4):1727–33 –Koohestanimobarhan S, Salami S, Imeni V, Mohammadi Z, Bayat O. Lipophilic statins antagonistically alter the major epithelial-to-mesenchymal transition signaling pathways in breast cancer stem-like cells via inhibition of the mevalonate pathway. J Cell Biochem. 2018 Sep 6.
Auranofin	RA	NFkB pathway inhibitor	–Raninga PV, Lee AC, Sinha D, Shih Y-Y, Mittal D, Makhale A, et al. Therapeutic cooperation between auranofin, a thioredoxin reductase inhibitor and anti-PD-L1 antibody for treatment of triple-negative breast cancer. Int J Cancer. 2019 May 15 –Hatem E, Azzi S, El Banna N, He T, Heneman-Masurel A, Vernis L, et al. Auranofin/Vitamin C: A Novel Drug Combination Targeting Triple-Negative Breast Cancer. J Natl Cancer Inst. 2018 Nov 20
Azithromycin	Bacterial infection, CAP, PID	Bacterial 50S ribosomal subunit inhibitor	
Bazedoxifene	Osteoporosis	selective estrogen receptor modulator (SERM)	–Fu S, Lin J. Blocking Interleukin-6 and Interleukin-8 Signaling Inhibits Cell Viability, Colony-forming Activity, and Cell Migration in Human Triple-negative Breast Cancer and Pancreatic Cancer Cells. Anticancer Res. 2018 Nov;38(11):6271–9 –Fu S, Chen X, Lo H-W, Lin J. Combined bazedoxifene and paclitaxel treatments inhibit cell viability, cell migration, colony formation, and tumor growth and induce apoptosis in breast cancer. Cancer Lett. 2019 Apr 28;448:11–9. –Tian J, Chen X, Fu S, Zhang R, Pan L, Cao Y, et al. Bazedoxifene is a novel IL-6/GP130 inhibitor for treating triple-negative breast cancer. Breast Cancer Res Treat. 2019 Jun;175(3):553–66
Bepridil	Hypertension and chronic stable angina	Calcium channel blocker	–Park S-H, Chung YM, Ma J, Yang Q, Berek JS, Hu MC-T. Pharmacological activation of FOXO3 suppresses triple-negative breast cancer in vitro and in vivo. Oncotarget. 2016 Jul 5;7(27):42110–25.
Calcitriol	Vitamin D deficiency	Vitamin D receptor agonist	–Martínez-Reza I, Díaz L, Barrera D, Segovia-Mendoza M, Pedraza-Sánchez S, Soca-Chafre G, et al. Calcitriol Inhibits the Proliferation of Triple-Negative Breast Cancer Cells through a Mechanism Involving the Proinflammatory Cytokines IL-1β and TNF-α. J Immunol Res. 2019;2019:6384278 –Zheng W, Cao L, Ouyang L, Zhang Q, Duan B, Zhou W, et al. Anticancer activity of 1,25-(OH)2D3 against human breast cancer cell lines by targeting Ras/MEK/ERK pathway. Onco Targets Ther. 2019;12:721–32 –Bijian K, Kaldre D, Wang T-T, Su J, Bouttier M, Boucher A, et al. Efficacy of hybrid vitamin D receptor agonist/histone deacetylase inhibitors in vitamin D-resistant triple-negative 4T1 breast cancer. J Steroid Biochem Mol Biol. 2018;177:135–9 –Bohl L, Guizzardi S, Rodríguez V, Hinrichsen L, Rozados V, Cremonezzi D, et al. Combined calcitriol and menadione reduces experimental murine triple negative breast tumor. Biomed Pharmacother. 2017 Oct;94:21–6 –Shan NL, Wahler J, Lee HJ, Bak MJ, Gupta SD, Maehr H, et al. Vitamin D compounds inhibit cancer stem-like cells and induce differentiation in triple negative breast cancer. J Steroid Biochem Mol Biol. 2017;173:122–9 –Thakkar A, Wang B, Picon-Ruiz M, Buchwald P, Ince TA. Vitamin D and androgen receptor-targeted therapy for triple-negative breast cancer. Breast Cancer Res Treat. 2016;157(1):77–90 –Richards SE, Weierstahl KA, Kelts JL. Vitamin D effect on growth and vitamin D metabolizing enzymes in triple-negative breast cancer. Anticancer Res. 2015 Feb;35(2):805–10 –Lopes N, Carvalho J, Durães C, Sousa B, Gomes M, Costa JL, et al. 1Alpha,25-dihydroxyvitamin D3 induces de novo E-cadherin expression in triple-negative breast cancer cells by CDH1-promoter demethylation. Anticancer Res. 2012 Jan;32(1):249–57
Carglumic acid	Hyperammonaemia in N-acetylglutamate synthase deficiency	Carbamoyl phosphate synthase activator	–Chen C-T, Chen Y-C, Yamaguchi H, Hung M-C. Carglumic acid promotes apoptosis and suppresses cancer cell proliferation in vitro and in vivo. Am J Cancer Res. 2015;5(12):3560–9
Celecoxib	OA, RA, JRA, AS, acute pain, primary dysmenorrhea	Cyclooxygenase inhibitor	–Ma Q, Gao Y, Wei D-F, Jiang N-H, Ding L, He X, et al. The effects of celecoxib on the proliferation and ultrastructural changes of MDA-MB-231 breast cancer cells. Ultrastruct Pathol. 2018 Jun;42(3):289–94 –Thomas S, Sharma N, Golden EB, Cho H, Agarwal P, Gaffney KJ, et al. Preferential killing of triple-negative breast cancer cells in vitro and in vivo when pharmacological aggravators of endoplasmic reticulum stress are combined with autophagy inhibitors. Cancer Lett. 2012 Dec 1;325(1):63–71
Chloroquine	Malaria, Extraintestinal Amebiasis	Antimalarial agent	–Liang DH, Choi DS, Ensor JE, Kaipparettu BA, Bass BL, Chang JC. The autophagy inhibitor chloroquine targets cancer stem cells in triple negative breast cancer by inducing mitochondrial damage and impairing DNA break repair. Cancer Lett. 2016 01;376(2):249–58 –Bouchard G, Therriault H, Geha S, Bérubé-Lauzière Y, Bujold R, Saucier C, et al. Stimulation of triple negative breast cancer cell migration and metastases formation is prevented by chloroquine in a pre-irradiated mouse model. BMC Cancer. 2016 10;16:361 –Tuomela J, Sandholm J, Kauppila JH, Lehenkari P, Harris KW, Selander KS. Chloroquine has tumor-inhibitory and tumor-promoting effects in triple-negative breast cancer. Oncol Lett. 2013 Dec;6(6):1665–72 –Chang C-T, Korivi M, Huang H-C, Thiyagarajan V, Lin K-Y, Huang P-J, et al. Inhibition of ROS production, autophagy or apoptosis signaling reversed the anticancer properties of Antrodia salmonea in triple-negative breast cancer (MDA-MB-231) cells. Food Chem Toxicol. 2017 May;103:1–17. –Rao R, Balusu R, Fiskus W, Mudunuru U, Venkannagari S, Chauhan L, et al. Combination of pan-histone deacetylase inhibitor and autophagy inhibitor exerts superior efficacy against triple-negative human breast cancer cells. Mol Cancer Ther. 2012 Apr;11(4):973–83. –Hu J, Zhang Y, Jiang X, Zhang H, Gao Z, Li Y, et al. ROS-mediated activation and mitochondrial translocation of CaMKII contributes to Drp1-dependent mitochondrial fission and apoptosis in triple-negative breast cancer cells by isorhamnetin and chloroquine. J Exp Clin Cancer Res. 2019 May 28;38(1):225. –Choi DS, Blanco E, Kim Y-S, Rodriguez AA, Zhao H, Huang TH-M, et al. Chloroquine eliminates cancer stem cells through deregulation of Jak2 and DNMT1. Stem Cells. 2014 Sep;32(9):2309–23 –Wang Z, Shi X, Li Y, Fan J, Zeng X, Xian Z, et al. Blocking autophagy enhanced cytotoxicity induced by recombinant human arginase in triple-negative breast cancer cells. Cell Death Dis. 2014 Dec 11;5:e1563 –Salaroglio IC, Gazzano E, Abdullrahman A, Mungo E, Castella B, Abd-Elrahman GEFA-E, et al. Increasing intratumor C/EBP-β LIP and nitric oxide levels overcome resistance to doxorubicin in triple negative breast cancer. J Exp Clin Cancer Res. 2018 Nov 27;37(1):286 –Thomas S, Sharma N, Golden EB, Cho H, Agarwal P, Gaffney KJ, et al. Preferential killing of triple-negative breast cancer cells in vitro and in vivo when pharmacological aggravators of endoplasmic reticulum stress are combined with autophagy inhibitors. Cancer Lett. 2012 Dec 1;325(1):63–71 –Lefort S, Joffre C, Kieffer Y, Givel A-M, Bourachot B, Zago G, et al. Inhibition of autophagy as a new means of improving chemotherapy efficiency in high-LC3B triple-negative breast cancers. Autophagy. 2014;10(12):2122–42 –Chen M, He M, Song Y, Chen L, Xiao P, Wan X, et al. The cytoprotective role of gemcitabine-induced autophagy associated with apoptosis inhibition in triple-negative MDA-MB-231 breast cancer cells. Int J Mol Med. 2014 Jul;34(1):276–82 –Abdel-Mohsen MA, Abdel Malak CA, El-Shafey ES. Influence of copper (I) nicotinate complex and autophagy modulation on doxorubicin-induced cytotoxicity in HCC1806 breast cancer cells. Adv Med Sci. 2019 Mar;64(1):202–9
Chlorpromazine	Psychotic disorders, nausea and vomiting, anxiety, hiccups	Dopamine receptor antagonist	–Zhao Y-Q, Yin Y-Q, Liu J, Wang G-H, Huang J, Zhu L-J, et al. Characterization of HJ-PI01 as a novel Pim-2 inhibitor that induces apoptosis and autophagic cell death in triple-negative human breast cancer. Acta Pharmacol Sin. 2016 Sep;37(9):1237–5
Cholecalciferol	Vitamin D deficiency		–Kutlehria S, Behl G, Patel K, Doddapaneni R, Vhora I, Chowdhury N, et al. Cholecalciferol-PEG Conjugate Based Nanomicelles of Doxorubicin for Treatment of Triple-Negative Breast Cancer. AAPS PharmSciTech. 2018 Feb;19(2):792–802.
Ciprofloxacin	Antibiotic	Bacterial DNA gyrase inhibitor	–Beberok A, Wrześniok D, Rok J, Rzepka Z, Respondek M, Buszman E. Ciprofloxacin triggers the apoptosis of human triple-negative breast cancer MDA-MB-231 cells via the p53/Bax/Bcl-2 signaling pathway. Int J Oncol. 2018 Mar 8
Clotrimazole	Fungal infections	Cytochrome P450 inhibitor|imidazoline receptor ligand	–Zhang P, Yang X, Yin Q, Yi J, Shen W, Zhao L, et al. Inhibition of SK4 Potassium Channels Suppresses Cell Proliferation, Migration and the Epithelial-Mesenchymal Transition in Triple-Negative Breast Cancer Cells. PLoS ONE. 2016;11(4):e0154471.
Colchicine	Gout	Microtubule inhibitor	–Lindamulage IK, Vu H-Y, Karthikeyan C, Knockleby J, Lee Y-F, Trivedi P, et al. Novel quinolone chalcones targeting colchicine-binding pocket kill multidrug-resistant cancer cells by inhibiting tubulin activity and MRP1 function. Sci Rep. 2017 31;7(1):10298.
Danazol	Endometriosis, fibrocystic breast disease, hereditary angioedema	Estrogen receptor antagonist|progesterone receptor agonist	–Deka SJ, Roy A, Ramakrishnan V, Manna D, Trivedi V. Danazol has potential to cause PKC translocation, cell cycle dysregulation, and apoptosis in breast cancer cells. Chem Biol Drug Des. 2017;89(6):953–63
Deferasirox	Acute iron intoxication, chronic iron overload	Chelating agent	–Tury S, Assayag F, Bonin F, Chateau-Joubert S, Servely J-L, Vacher S, et al. The iron chelator deferasirox synergises with chemotherapy to treat triple-negative breast cancers. J Pathol. 2018 Sep;246(1):103–14
Deferiprone	Iron overload in thalassemia major	Chelating agent	–Knickle A, Fernando W, Greenshields AL, Rupasinghe HPV, Hoskin DW. Myricetin-induced apoptosis of triple-negative breast cancer cells is mediated by the iron-dependent generation of reactive oxygen species from hydrogen peroxide. Food Chem Toxicol. 2018 Aug;118:154–67
Digitoxin	Congestive HF, atrial fibrillation, atrial flutter, PAT, cardiogenic shock	ATPase inhibitor	–Kulkarni YM, Yakisich JS, Azad N, Venkatadri R, Kaushik V, O’Doherty G, et al. Anti-tumorigenic effects of a novel digitoxin derivative on both estrogen receptor-positive and triple-negative breast cancer cells. Tumour Biol. 2017 Jun;39(6):1010428317705331
Digoxin	Heart failure, atrial fibrillation	ATPase inhibitor	–Samanta D, Gilkes DM, Chaturvedi P, Xiang L, Semenza GL. Hypoxia-inducible factors are required for chemotherapy resistance of breast cancer stem cells. Proc Natl Acad Sci USA. 2014 Dec 16;111(50):E5429-5438
Dipyridamole	Thromboembolism Prophylaxis Post-Cardiac Valve Replacement	Phosphodiesterase inhibitor	–Spano D, Marshall J-C, Marino N, De Martino D, Romano A, Scoppettuolo MN, et al. Dipyridamole prevents triple-negative breast-cancer progression. Clin Exp Metastasis. 2013 Jan;30(1):47–68
Disulfiram	Chronic alcoholism	Aldehyde dehydrogenase inhibitor|DNA methyltransferase inhibitor|TRPV agonist	–Kim JY, Lee N, Kim Y-J, Cho Y, An H, Oh E, et al. Disulfiram induces anoikis and suppresses lung colonization in triple-negative breast cancer via calpain activation. Cancer Lett. 2017 01;386:151–60. –Kim Y-J, Kim JY, Lee N, Oh E, Sung D, Cho T-M, et al. Disulfiram suppresses cancer stem-like properties and STAT3 signaling in triple-negative breast cancer cells. Biochem Biophys Res Commun. 2017 May 13;486(4):1069–76. –Robinson TJW, Pai M, Liu JC, Vizeacoumar F, Sun T, Egan SE, et al. High-throughput screen identifies disulfiram as a potential therapeutic for triple-negative breast cancer cells: interaction with IQ motif-containing factors. Cell Cycle. 2013 Sep 15;12(18):3013–24. –Liu P, Kumar IS, Brown S, Kannappan V, Tawari PE, Tang JZ, et al. Disulfiram targets cancer stem-like cells and reverses resistance and cross-resistance in acquired paclitaxel-resistant triple-negative breast cancer cells. Br J Cancer. 2013 Oct 1;109(7):1876–85. –Wu L, Meng F, Dong L, Block CJ, Mitchell AV, Wu J, et al. Disulfiram and BKM120 in Combination with Chemotherapy Impede Tumor Progression and Delay Tumor Recurrence in Tumor Initiating Cell-Rich TNBC. Sci Rep. 2019 Jan 18;9(1):236.
Doxycycline	Respiratory/urinary tract/ophtalmic infection	Metalloproteinase inhibitor	–Lin C-C, Lo M-C, Moody RR, Stevers NO, Tinsley SL, Sun D. Doxycycline targets aldehyde dehydrogenase‑positive breast cancer stem cells. Oncol Rep. 2018 Jun;39(6):3041–7.
Dutasteride	Benign prostatic hyperplasia	5 alpha reductase inhibitor	–von Wahlde M-K, Hülsewig C, Ruckert C, Götte M, Kiesel L, Bernemann C. The anti-androgen drug dutasteride renders triple negative breast cancer cells more sensitive to chemotherapy via inhibition of HIF-1α-/VEGF-signaling. Gynecol Endocrinol. 2015 Feb;31(2):160–4.
Esomeprazole	Antacid	ATPase inhibitor	–Goh W, Sleptsova-Freidrich I, Petrovic N. Use of proton pump inhibitors as adjunct treatment for triple-negative breast cancers. An introductory study. J Pharm Pharm Sci. 2014;17(3):439–46
Fasudil	Vasodilator	Rho associated kinase inhibitor	–Guerra FS, Oliveira RG de, Fraga CAM, Mermelstein CDS, Fernandes PD. ROCK inhibition with Fasudil induces beta-catenin nuclear translocation and inhibits cell migration of MDA-MB 231 human breast cancer cells. Sci Rep. 2017 20;7(1):13723.
Fenofibrate	Hyperlipidemia	PPAR receptor agonist	–Li T, Zhang Q, Zhang J, Yang G, Shao Z, Luo J, et al. Fenofibrate induces apoptosis of triple-negative breast cancer cells via activation of NF-κB pathway. BMC Cancer. 2014 Feb 16;14:96.
Fingolimod	Multiple Sclerosis	Immunosuppressant|sphingosine phosphate receptor agonist	–Martin JL, Julovi SM, Lin MZ, de Silva HC, Boyle FM, Baxter RC. Inhibition of basal-like breast cancer growth by FTY720 in combination with epidermal growth factor receptor kinase blockade. Breast Cancer Res. 2017 Aug 4;19(1):90. –Alshaker H, Wang Q, Srivats S, Chao Y, Cooper C, Pchejetski D. New FTY720-docetaxel nanoparticle therapy overcomes FTY720-induced lymphopenia and inhibits metastatic breast tumour growth. Breast Cancer Res Treat. 2017 Oct;165(3):531–43 –Hait NC, Avni D, Yamada A, Nagahashi M, Aoyagi T, Aoki H, et al. The phosphorylated prodrug FTY720 is a histone deacetylase inhibitor that reactivates ERα expression and enhances hormonal therapy for breast cancer. Oncogenesis. 2015 Jun 8;4:e156
Flubendazole	Parasitic infection	Tubulin polymerisation inhibitor	–Oh E, Kim Y-J, An H, Sung D, Cho T-M, Farrand L, et al. Flubendazole elicits anti-metastatic effects in triple-negative breast cancer via STAT3 inhibition. Int J Cancer. 2018 15;143(8):1978–93 –Zhang L, Guo M, Li J, Zheng Y, Zhang S, Xie T, et al. Systems biology-based discovery of a potential Atg4B agonist (Flubendazole) that induces autophagy in breast cancer. Mol Biosyst. 2015 Nov;11(11):2860–6.
Fluoxetine	Depression	Selective serotonin reuptake inhibitor (SSRI)	–Sun D, Zhu L, Zhao Y, Jiang Y, Chen L, Yu Y, et al. Fluoxetine induces autophagic cell death via eEF2K-AMPK-mTOR-ULK complex axis in triple negative breast cancer. Cell Prolif. 2018 Apr;51(2):e12402. –Bowie M, Pilie P, Wulfkuhle J, Lem S, Hoffman A, Desai S, et al. Fluoxetine induces cytotoxic endoplasmic reticulum stress and autophagy in triple negative breast cancer. World J Clin Oncol. 2015 Dec 10;6(6):299–311
Fluvastatin	Hyperlipidemia	HMGCR inhibitor	–Bhardwaj A, Singh H, Trinidad CM, Albarracin CT, Hunt KK, Bedrosian I. The isomiR-140-3p-regulated mevalonic acid pathway as a potential target for prevention of triple negative breast cancer. Breast Cancer Res. 2018 11;20(1):150.
Ganciclovir	Anti-viral	DNA polymerase inhibitor	–Castillo-Rodríguez RA, Arango-Rodríguez ML, Escobedo L, Hernandez-Baltazar D, Gompel A, Forgez P, et al. Suicide HSVtk gene delivery by neurotensin-polyplex nanoparticles via the bloodstream and GCV Treatment specifically inhibit the growth of human MDA-MB-231 triple negative breast cancer tumors xenografted in athymic mice. PLoS ONE. 2014;9(5):e97151 –Devulapally R, Lee T, Barghava-Shah A, Sekar TV, Foygel K, Bachawal SV, et al. Ultrasound-guided delivery of thymidine kinase-nitroreductase dual therapeutic genes by PEGylated-PLGA/PIE nanoparticles for enhanced triple negative breast cancer therapy. Nanomedicine (Lond). 2018;13(9):1051–66
Hydralazine	Hypertension	Vasodilator	–Jiang Y, Huang Y, Cheng C, Lu W, Zhang Y, Liu X, et al. Combination of thiazolidinedione and hydralazine suppresses proliferation and induces apoptosis by PPARγ up-expression in MDA-MB-231 cells. Exp Mol Pathol. 2011 Dec;91(3):768–74
Hydroxychloroquine	Malaria		–Chittaranjan S, Bortnik S, Dragowska WH, Xu J, Abeysundara N, Leung A, et al. Autophagy inhibition augments the anticancer effects of epirubicin treatment in anthracycline-sensitive and -resistant triple-negative breast cancer. Clin Cancer Res. 2014 Jun 15;20(12):3159–73
Indomethacin	Analgesia	Cyclooxygenase inhibitor	–Basudhar D, Glynn SA, Greer M, Somasundaram V, No JH, Scheiblin DA, et al. Coexpression of NOS2 and COX2 accelerates tumor growth and reduces survival in estrogen receptor-negative breast cancer. Proc Natl Acad Sci USA. 2017 05;114(49):13030–5.
Ivermectin	Parasitic infection	Benzodiazepine receptor agonist	–Kwon Y-J, Petrie K, Leibovitch BA, Zeng L, Mezei M, Howell L, et al. Selective Inhibition of SIN3 Corepressor with Avermectins as a Novel Therapeutic Strategy in Triple-Negative Breast Cancer. Mol Cancer Ther. 2015 Aug;14(8):1824–36
Leflunomide	Arthritis	D Dihydroorotate dehydrogenase inhibitor|PDGFR tyrosine kinase receptor inhibitor	–Brown KK, Spinelli JB, Asara JM, Toker A. Adaptive Reprogramming of De Novo Pyrimidine Synthesis Is a Metabolic Vulnerability in Triple-Negative Breast Cancer. Cancer Discov. 2017;7(4):391–9. –Jin U-H, Lee S-O, Pfent C, Safe S. The aryl hydrocarbon receptor ligand omeprazole inhibits breast cancer cell invasion and metastasis. BMC Cancer. 2014 Jul 9;14:498.
Losartan	Hypertension	Angiotensin receptor antagonist	–Hu C, Liu X, Ran W, Meng J, Zhai Y, Zhang P, et al. Regulating cancer associated fibroblasts with losartan-loaded injectable peptide hydrogel to potentiate chemotherapy in inhibiting growth and lung metastasis of triple negative breast cancer. Biomaterials. 2017 Nov;144:60–72
Lovastatin	Hyperlipidemia	HMGCR inhibitor	–Song L, Tao X, Lin L, Chen C, Yao H, He G, et al. Cerasomal Lovastatin Nanohybrids for Efficient Inhibition of Triple-Negative Breast Cancer Stem Cells To Improve Therapeutic Efficacy. ACS Appl Mater Interfaces. 2018 Feb 28;10(8):7022–30 –Zhang N, Liang X, Gao C, Chen M, Zhou Y, Krueger CJ, et al. Loading Lovastatin into Camptothecin-Floxuridine Conjugate Nanocapsules for Enhancing Anti-metastatic Efficacy of Cocktail Chemotherapy on Triple-negative Breast Cancer. ACS Appl Mater Interfaces. 2018 Sep 5;10(35):29385–97. –Lin Z, Zhang Z, Jiang X, Kou X, Bao Y, Liu H, et al. Mevastatin blockade of autolysosome maturation stimulates LBH589-induced cell death in triple-negative breast cancer cells. Oncotarget. 2017 Mar 14;8(11):17833–48 –Koohestanimobarhan S, Salami S, Imeni V, Mohammadi Z, Bayat O. Lipophilic statins antagonistically alter the major epithelial-to-mesenchymal transition signaling pathways in breast cancer stem-like cells via inhibition of the mevalonate pathway. J Cell Biochem. 2018 Sep 6
Maraviroc	Anti-retroviral	CC chemokine receptor antagonist	–Norton K-A, Wallace T, Pandey NB, Popel AS. An agent-based model of triple-negative breast cancer: the interplay between chemokine receptor CCR5 expression, cancer stem cells, and hypoxia. BMC Syst Biol. 2017 Jul 11;11(1):68 –in K, Pandey NB, Popel AS. Simultaneous blockade of IL-6 and CCL5 signaling for synergistic inhibition of triple-negative breast cancer growth and metastasis. Breast Cancer Res. 2018 14;20(1):54.
Mebendazole	Parasitic infection	Tubulin polymerisation inhibitor	–Zhang L, Bochkur Dratver M, Yazal T, Dong K, Nguyen A, Yu G, et al. Mebendazole Potentiates Radiation Therapy in Triple-Negative Breast Cancer. Int J Radiat Oncol Biol Phys. 2019 Jan 1;103(1):195–207
Melatonin	Insomnia	Melatonin receptor agonist|nitric oxide synthase inhibitor	–Kim T-H, Cho S-G. Melatonin-induced KiSS1 expression inhibits triple-negative breast cancer cell invasiveness. Oncol Lett. 2017 Aug;14(2):2511–6 –Marques JHM, Mota AL, Oliveira JG, Lacerda JZ, Stefani JP, Ferreira LC, et al. Melatonin restrains angiogenic factors in triple-negative breast cancer by targeting miR-152-3p: In vivo and in vitro studies. Life Sci. 2018 Sep 1;208:131–8 –Lacerda JZ, Ferreira LC, Lopes BC, Aristizábal-Pachón AF, Bajgelman MC, Borin TF, et al. Therapeutic Potential of Melatonin in the Regulation of MiR-148a-3p and Angiogenic Factors in Breast Cancer. Microrna. 2019;8(3):237–47 –Jardim-Perassi BV, Arbab AS, Ferreira LC, Borin TF, Varma NRS, Iskander ASM, et al. Effect of melatonin on tumor growth and angiogenesis in xenograft model of breast cancer. PLoS ONE. 2014;9(1):e85311 –Jardim-Perassi BV, Alexandre PA, Sonehara NM, de Paula-Junior R, Reis Júnior O, Fukumasu H, et al. RNA-Seq transcriptome analysis shows anti-tumor actions of melatonin in a breast cancer xenograft model. Sci Rep. 2019 Jan 30;9(1):966
Metformin	Diabetes	Insulin sensitizer	–Cheng G, Zielonka J, Hardy M, Ouari O, Chitambar CR, Dwinell MB, et al. Synergistic inhibition of tumor cell proliferation by metformin and mito-metformin in the presence of iron chelators. Oncotarget. 2019 May 28;10(37):3518–32 –Han Y, Li C-W, Hsu J-M, Hsu JL, Chan L-C, Tan X, et al. Metformin reverses PARP inhibitors-induced epithelial-mesenchymal transition and PD-L1 upregulation in triple-negative breast cancer. Am J Cancer Res. 2019;9(4):800–15 –Varghese S, Samuel SM, Varghese E, Kubatka P, Büsselberg D. High Glucose Represses the Anti-Proliferative and Pro-Apoptotic Effect of Metformin in Triple Negative Breast Cancer Cells. Biomolecules. 2019 08;9(1). –Bhardwaj A, Singh H, Trinidad CM, Albarracin CT, Hunt KK, Bedrosian I. The isomiR-140-3p-regulated mevalonic acid pathway as a potential target for prevention of triple negative breast cancer. Breast Cancer Res. 2018 11;20(1):150. –Wahdan-Alaswad RS, Edgerton SM, Salem HS, Thor AD. Metformin Targets Glucose Metabolism in Triple Negative Breast Cancer. J Oncol Transl Res. 2018;4(1). –Amaral I, Silva C, Correia-Branco A, Martel F. Effect of metformin on estrogen and progesterone receptor-positive (MCF-7) and triple-negative (MDA-MB-231) breast cancer cells. Biomed Pharmacother. 2018 Jun;102:94–101. –Amaral MEA, Nery LR, Leite CE, de Azevedo Junior WF, Campos MM. Pre-clinical effects of metformin and aspirin on the cell lines of different breast cancer subtypes. Invest New Drugs. 2018;36(5):782–96. –Shi P, Liu W, Tala null, Wang H, Li F, Zhang H, et al. Metformin suppresses triple-negative breast cancer stem cells by targeting KLF5 for degradation. Cell Discov. 2017;3:17010. –Wokoun U, Hellriegel M, Emons G, Gründker C. Co-treatment of breast cancer cells with pharmacologic doses of 2-deoxy-D-glucose and metformin: Starving tumors. Oncol Rep. 2017 Apr;37(4):2418–24. –Strekalova E, Malin D, Rajanala H, Cryns VL. Metformin sensitizes triple-negative breast cancer to proapoptotic TRAIL receptor agonists by suppressing XIAP expression. Breast Cancer Res Treat. 2017 Jun;163(3):435–47. –García-Castillo V, López-Urrutia E, Villanueva-Sánchez O, Ávila-Rodríguez MÁ, Zentella-Dehesa A, Cortés-González C, et al. Targeting Metabolic Remodeling in Triple Negative Breast Cancer in a Murine Model. J Cancer. 2017;8(2):178–89. –Rico M, Baglioni M, Bondarenko M, Laluce NC, Rozados V, André N, et al. Metformin and propranolol combination prevents cancer progression and metastasis in different breast cancer models. Oncotarget. 2017 Jan 10;8(2):2874–89. –Wahdan-Alaswad R, Harrell JC, Fan Z, Edgerton SM, Liu B, Thor AD. Metformin attenuates transforming growth factor beta (TGF-β) mediated oncogenesis in mesenchymal stem-like/claudin-low triple negative breast cancer. Cell Cycle. 2016;15(8):1046–59. –Talarico G, Orecchioni S, Dallaglio K, Reggiani F, Mancuso P, Calleri A, et al. Aspirin and atenolol enhance metformin activity against breast cancer by targeting both neoplastic and microenvironment cells. Sci Rep. 2016 Jan 5;6:18673. –Marinello PC, da Silva TNX, Panis C, Neves AF, Machado KL, Borges FH, et al. Mechanism of metformin action in MCF-7 and MDA-MB-231 human breast cancer cells involves oxidative stress generation, DNA damage, and transforming growth factor β1 induction. Tumour Biol. 2016 Apr;37(4):5337–46.
Methimazole	Hyperthyroidism	Antithyroid agent	–Noori MS, O’Brien JD, Champa ZJ, Deosarkar SP, Lanier OL, Qi C, et al. Phenylmethimazole and a thiazole derivative of phenylmethimazole inhibit IL-6 expression by triple negative breast cancer cells. Eur J Pharmacol. 2017 May 15;803:130–7.
Mifepristone	Abortifacient	Glucocorticoid receptor antagonist|progesterone receptor antagonist	–Liu R, Shi P, Nie Z, Liang H, Zhou Z, Chen W, et al. Mifepristone Suppresses Basal Triple-Negative Breast Cancer Stem Cells by Down-regulating KLF5 Expression. Theranostics. 2016;6(4):533–44. –Skor MN, Wonder EL, Kocherginsky M, Goyal A, Hall BA, Cai Y, et al. Glucocorticoid receptor antagonism as a novel therapy for triple-negative breast cancer. Clin Cancer Res. 2013 Nov 15;19(22):6163–72.
Minocycline	Antibiotic	Bacterial 30S ribosomal subunit inhibitor	–Himmel LE, Lustberg MB, DeVries AC, Poi M, Chen C-S, Kulp SK. Minocycline, a putative neuroprotectant, co-administered with doxorubicin-cyclophosphamide chemotherapy in a xenograft model of triple-negative breast cancer. Exp Toxicol Pathol. 2016 Oct;68(9):505–15.
Montelukast	Allergies	Leukotriene receptor antagonist	–Suknuntha K, Yubolphan R, Krueaprasertkul K, Srihirun S, Sibmooh N, Vivithanaporn P. Leukotriene Receptor Antagonists Inhibit Mitogenic Activity in Triple Negative Breast Cancer Cells. Asian Pac J Cancer Prev. 2018 Mar 27;19(3):833–7.
Nelfinavir	Anti-retroviral	HIV protease inhibitor	–Thomas S, Sharma N, Golden EB, Cho H, Agarwal P, Gaffney KJ, et al. Preferential killing of triple-negative breast cancer cells in vitro and in vivo when pharmacological aggravators of endoplasmic reticulum stress are combined with autophagy inhibitors. Cancer Lett. 2012 Dec 1;325(1):63–71.
Niclosamide	Parasitic infection	DNA replication inhibitor|STAT inhibitor	–Yin L, Gao Y, Zhang X, Wang J, Ding D, Zhang Y, et al. Niclosamide sensitizes triple-negative breast cancer cells to ionizing radiation in association with the inhibition of Wnt/β-catenin signaling. Oncotarget. 2016 Jul 5;7(27):42126–38. –Liu J, Chen X, Ward T, Pegram M, Shen K. Combined niclosamide with cisplatin inhibits epithelial-mesenchymal transition and tumor growth in cisplatin-resistant triple-negative breast cancer. Tumour Biol. 2016 Jul;37(7):9825–35. –Lu L, Dong J, Wang L, Xia Q, Zhang D, Kim H, et al. Activation of STAT3 and Bcl-2 and reduction of reactive oxygen species (ROS) promote radioresistance in breast cancer and overcome of radioresistance with niclosamide. Oncogene. 2018;37(39):5292–304. –Pindiprolu SKSS, Chintamaneni PK, Krishnamurthy PT, Ratna Sree Ganapathineedi K. Formulation-optimisation of solid lipid nanocarrier system of STAT3 inhibitor to improve its activity in triple negative breast cancer cells. Drug Dev Ind Pharm. 2019 Feb;45(2):304–13.
Nicotinamide	Niacin Deficiency, Skin cancer chemoprevention	Protein synthesis stimulant	–Kim JY, Lee H, Woo J, Yue W, Kim K, Choi S, et al. Reconstruction of pathway modification induced by nicotinamide using multi-omic network analyses in triple negative breast cancer. Sci Rep. 2017 14;7(1):3466.
Nimodipine	Hypertension	Calcium channel blocker	–Jin U-H, Lee S-O, Pfent C, Safe S. The aryl hydrocarbon receptor ligand omeprazole inhibits breast cancer cell invasion and metastasis. BMC Cancer. 2014 Jul 9;14:498.
Noscapine	Anti-tussive	Bradykinin receptor antagonist|tubulin polymerisation inhibitor	–Doddapaneni R, Patel K, Chowdhury N, Singh M. Noscapine chemosensitization enhances docetaxel anticancer activity and nanocarrier uptake in triple negative breast cancer. Exp Cell Res. 2016 01;346(1):65–73. –Chougule MB, Patel AR, Jackson T, Singh M. Antitumor activity of Noscapine in combination with Doxorubicin in triple negative breast cancer. PLoS ONE. 2011 Mar 15;6(3):e17733. –Doddapaneni R, Patel K, Chowdhury N, Singh M. Reversal of drug-resistance by noscapine chemo-sensitization in docetaxel resistant triple negative breast cancer. Sci Rep. 2017 Nov 20;7(1):15824.
Omega 3	Hyperlipidemia		–Pizato N, Luzete BC, Kiffer LFMV, Corrêa LH, de Oliveira Santos I, Assumpção JAF, et al. Omega-3 docosahexaenoic acid induces pyroptosis cell death in triple-negative breast cancer cells. Sci Rep. 2018 31;8(1):1952. –Torres-Adorno AM, Vitrac H, Qi Y, Tan L, Levental KR, Fan Y-Y, et al. Eicosapentaenoic acid in combination with EPHA2 inhibition shows efficacy in preclinical models of triple-negative breast cancer by disrupting cellular cholesterol efflux. Oncogene. 2019;38(12):2135–50. –Pizato N, Kiffer LFMV, Luzete BC, Assumpção JAF, Correa LH, Melo HAB de, et al. Omega 3-DHA and Delta-Tocotrienol Modulate Lipid Droplet Biogenesis and Lipophagy in Breast Cancer Cells: the Impact in Cancer Aggressiveness. Nutrients. 2019 May 28;11(6). –Pogash TJ, El-Bayoumy K, Amin S, Gowda K, de Cicco RL, Barton M, et al. Oxidized derivative of docosahexaenoic acid preferentially inhibit cell proliferation in triple negative over luminal breast cancer cells. In Vitro Cell Dev Biol Anim. 2015 Feb;51(2):121–7 –Blanckaert V, Kerviel V, Lépinay A, Joubert-Durigneux V, Hondermarck H, Chénais B. Docosahexaenoic acid inhibits the invasion of MDA-MB-231 breast cancer cells through upregulation of cytokeratin-1. Int J Oncol. 2015;46(6):2649–55.
Omeprazole	Antacid	ATPase inhibitor	–Jin U-H, Lee S-O, Pfent C, Safe S. The aryl hydrocarbon receptor ligand omeprazole inhibits breast cancer cell invasion and metastasis. BMC Cancer. 2014 Jul 9;14:498.
Orlistat	Obesity	Lipase inhibitor	–Paulmurugan R, Bhethanabotla R, Mishra K, Devulapally R, Foygel K, Sekar TV, et al. Folate Receptor-Targeted Polymeric Micellar Nanocarriers for Delivery of Orlistat as a Repurposed Drug against Triple-Negative Breast Cancer. Mol Cancer Ther. 2016 Feb;15(2):221–31 –Bhargava-Shah A, Foygel K, Devulapally R, Paulmurugan R. Orlistat and antisense-miRNA-loaded PLGA-PEG nanoparticles for enhanced triple negative breast cancer therapy. Nanomedicine (Lond). 2016 Feb;11(3):235–47.
Penfluridol	Psychotic disorders	T-type calcium channel blocker	–Ranjan A, Gupta P, Srivastava SK. Penfluridol: An Antipsychotic Agent Suppresses Metastatic Tumor Growth in Triple-Negative Breast Cancer by Inhibiting Integrin Signaling Axis. Cancer Res. 2016 Feb 15;76(4):877–90.
Pentamidine	Parasitic infection	Anti-pneumocystis agent	–Her S, Cui L, Bristow RG, Allen C. Dual Action Enhancement of Gold Nanoparticle Radiosensitization by Pentamidine in Triple Negative Breast Cancer. Radiat Res. 2016;185(5):549–62
Pentoxifylline	Peripheral artery disease	Phosphodiesterase inhibitor	–Castellanos-Esparza YC, Wu S, Huang L, Buquet C, Shen R, Sanchez-Gonzalez B, et al. Synergistic promoting effects of pentoxifylline and simvastatin on the apoptosis of triple-negative MDA-MB-231 breast cancer cells. Int J Oncol. 2018 Apr;52(4):1246–54
Pirfenidone	Anti-fibrotic	TGF beta receptor inhibitor	–Brooks D, Zimmer A, Wakefield L, Lyle LT, Difilippantonio S, Tucci FC, et al. Limited fibrosis accompanies triple-negative breast cancer metastasis in multiple model systems and is not a preventive target. Oncotarget. 2018 May 4;9(34):23462–81. –Takai K, Le A, Weaver VM, Werb Z. Targeting the cancer-associated fibroblasts as a treatment in triple-negative breast cancer. Oncotarget. 2016 Dec 13;7(50):82889–901 –Qi X, Yin N, Ma S, Lepp A, Tang J, Jing W, et al. p38γ MAPK Is a Therapeutic Target for Triple-Negative Breast Cancer by Stimulation of Cancer Stem-Like Cell Expansion. Stem Cells. 2015 Sep;33(9):2738–47.
Propranolol	Hypertension	Adrenergic receptor antagonist	–Rico M, Baglioni M, Bondarenko M, Laluce NC, Rozados V, André N, et al. Metformin and propranolol combination prevents cancer progression and metastasis in different breast cancer models. Oncotarget. 2017 Jan 10;8(2):2874–89. –Choy C, Raytis JL, Smith DD, Duenas M, Neman J, Jandial R, et al. Inhibition of β2-adrenergic receptor reduces triple-negative breast cancer brain metastases: The potential benefit of perioperative β-blockade. Oncol Rep. 2016 Jun;35(6):3135–42. –Pasquier E, Ciccolini J, Carre M, Giacometti S, Fanciullino R, Pouchy C, et al. Propranolol potentiates the anti-angiogenic effects and anti-tumor efficacy of chemotherapy agents: implication in breast cancer treatment. Oncotarget. 2011 Oct;2(10):797–809. –Xie W-Y, He R-H, Zhang J, He Y-J, Wan Z, Zhou C-F, et al. β‑blockers inhibit the viability of breast cancer cells by regulating the ERK/COX‑2 signaling pathway and the drug response is affected by ADRB2 single‑nucleotide polymorphisms. Oncol Rep. 2019 Jan;41(1):341–50.
Pyrimethamine	Parasitic infection	Dihydrofolate reductase inhibitor	–Egusquiaguirre SP, Yeh JE, Walker SR, Liu S, Frank DA. The STAT3 Target Gene TNFRSF1A Modulates the NF-κB Pathway in Breast Cancer Cells. Neoplasia. 2018;20(5):489–98.
Riluzole	ALS	Glutamate inhibitor	–Speyer CL, Smith JS, Banda M, DeVries JA, Mekani T, Gorski DH. Metabotropic glutamate receptor-1: a potential therapeutic target for the treatment of breast cancer. Breast Cancer Res Treat. 2012 Apr;132(2):565–73. –Speyer CL, Nassar MA, Hachem AH, Bukhsh MA, Jafry WS, Khansa RM, et al. Riluzole mediates anti-tumor properties in breast cancer cells independent of metabotropic glutamate receptor-1. Breast Cancer Res Treat. 2016;157(2):217–28. –Speyer CL, Bukhsh MA, Jafry WS, Sexton RE, Bandyopadhyay S, Gorski DH. Riluzole synergizes with paclitaxel to inhibit cell growth and induce apoptosis in triple-negative breast cancer. Breast Cancer Res Treat. 2017 Nov;166(2):407–19.
Simvastatin	Hyperlipidemia	HMGCR inhibitor	–Kou X, Yang Y, Jiang X, Liu H, Sun F, Wang X, et al. Vorinostat and Simvastatin have synergistic effects on triple-negative breast cancer cells via abrogating Rab7 prenylation. Eur J Pharmacol. 2017 Oct 15;813:161–71. –Wolfe AR, Debeb BG, Lacerda L, Larson R, Bambhroliya A, Huang X, Bertucci F, Finetti P, Birnbaum D, Van Laere S, Diagaradjan P, Ruffell B, Trenton NJ, Chu K, Hittelman W, Diehl M, Levental I, Ueno NT, Woodward WA. Simvastatin prevents triple-negative breast cancer metastasis in pre-clinical models through regulation of FOXO3a. Breast Cancer Res Treat. 2015 Dec;154(3):495-508 –Kou X, Jiang X, Liu H, Wang X, Sun F, Han J, et al. Simvastatin functions as a heat shock protein 90 inhibitor against triple-negative breast cancer. Cancer Sci. 2018 Oct;109(10):3272–84. –Jung HH, Lee S-H, Kim J-Y, Ahn JS, Park YH, Im Y-H. Statins affect ETS1-overexpressing triple-negative breast cancer cells by restoring DUSP4 deficiency. Sci Rep. 2016 08;6:33035 –Sulaiman A, McGarry S, Li L, Jia D, Ooi S, Addison C, et al. Dual inhibition of Wnt and Yes-associated protein signaling retards the growth of triple-negative breast cancer in both mesenchymal and epithelial states. Mol Oncol. 2018;12(4):423–40. –Lacerda L, Reddy JP, Liu D, Larson R, Li L, Masuda H, Brewer T, Debeb BG, Xu W, Hortobágyi GN, Buchholz TA, Ueno NT, Woodward WA. Simvastatin radiosensitizes differentiated and stem-like breast cancer cell lines and is associated with improved local control in inflammatory breast cancer patients treated with postmastectomy radiation. Stem Cells Transl Med. 2014 Jul;3(7):849-56. –Castellanos-Esparza YC, Wu S, Huang L, Buquet C, Shen R, Sanchez-Gonzalez B, et al. Synergistic promoting effects of pentoxifylline and simvastatin on the apoptosis of triple-negative MDA-MB-231 breast cancer cells. Int J Oncol. 2018 Apr;52(4):1246–54. –Abdoul-Azize S, Buquet C, Li H, Picquenot J-M, Vannier J-P. Integration of Ca2+ signaling regulates the breast tumor cell response to simvastatin and doxorubicin. Oncogene. 2018;37(36):4979–93. –Kanugula AK, Gollavilli PN, Vasamsetti SB, Karnewar S, Gopoju R, Ummanni R, et al. Statin-induced inhibition of breast cancer proliferation and invasion involves attenuation of iron transport: intermediacy of nitric oxide and antioxidant defence mechanisms. FEBS J. 2014 Aug;281(16):3719–38. –Park YH, Jung HH, Ahn JS, Im Y-H. Statin induces inhibition of triple negative breast cancer (TNBC) cells via PI3K pathway. Biochem Biophys Res Commun. 2013 Sep 20;439(2):275–9. –Koohestanimobarhan S, Salami S, Imeni V, Mohammadi Z, Bayat O. Lipophilic statins antagonistically alter the major epithelial-to-mesenchymal transition signaling pathways in breast cancer stem-like cells via inhibition of the mevalonate pathway. J Cell Biochem. 2018 Sep 6;
Sodium Bicarbonate	Relief of wind and griping pains		–Abumanhal-Masarweh H, Koren L, Zinger A, Yaari Z, Krinsky N, Kaneti G, et al. Sodium bicarbonate nanoparticles modulate the tumor pH and enhance the cellular uptake of doxorubicin. J Control Release. 2019 Feb 28;296:1–13.
Sulfasalazine	Rheumatoid arthritis; ulcerative colitis; active Crohn's Disease.	Cyclooxygenase inhibitor	–Hasegawa M, Takahashi H, Rajabi H, Alam M, Suzuki Y, Yin L, et al. Functional interactions of the cystine/glutamate antiporter, CD44v and MUC1-C oncoprotein in triple-negative breast cancer cells. Oncotarget. 2016 Mar 15;7(11):11756–69. –Timmerman LA, Holton T, Yuneva M, Louie RJ, Padró M, Daemen A, et al. Glutamine sensitivity analysis identifies the xCT antiporter as a common triple-negative breast tumor therapeutic target. Cancer Cell. 2013 Oct 14;24(4):450–65
Thioridazine	Psychotic disorders	Dopamine receptor antagonist	–Tegowski M, Fan C, Baldwin AS. Thioridazine inhibits self-renewal in breast cancer cells via DRD2-dependent STAT3 inhibition, but induces a G1 arrest independent of DRD2. J Biol Chem. 2018 12;293(41):15977–90. –Goyette M-A, Cusseddu R, Elkholi I, Abu-Thuraia A, El-Hachem N, Haibe-Kains B, et al. AXL knockdown gene signature reveals a drug repurposing opportunity for a class of antipsychotics to reduce growth and metastasis of triple-negative breast cancer. Oncotarget. 2019 Mar 12;10(21):2055–67
Tigecycline	Infections	Bacterial 30S ribosomal subunit inhibitor	–Jones RA, Robinson TJ, Liu JC, Shrestha M, Voisin V, Ju Y, et al. RB1 deficiency in triple-negative breast cancer induces mitochondrial protein translation. J Clin Invest. 2016 03;126(10):3739–57
Tocilizumab	Rheumatoid arthritis		–Jin K, Pandey NB, Popel AS. Simultaneous blockade of IL-6 and CCL5 signaling for synergistic inhibition of triple-negative breast cancer growth and metastasis. Breast Cancer Res. 2018 14;20(1):54. –Weng Y-S, Tseng H-Y, Chen Y-A, Shen P-C, Al Haq AT, Chen L-M, et al. MCT-1/miR-34a/IL-6/IL-6R signaling axis promotes EMT progression, cancer stemness and M2 macrophage polarization in triple-negative breast cancer. Mol Cancer. 2019 18;18(1):42.
Trifluoperazine	Psychotic disorders	Dopamine receptor antagonist	–Goyette M-A, Cusseddu R, Elkholi I, Abu-Thuraia A, El-Hachem N, Haibe-Kains B, et al. AXL knockdown gene signature reveals a drug repurposing opportunity for a class of antipsychotics to reduce growth and metastasis of triple-negative breast cancer. Oncotarget. 2019 Mar 12;10(21):2055–67. –Feng Z, Xia Y, Gao T, Xu F, Lei Q, Peng C, et al. The antipsychotic agent trifluoperazine hydrochloride suppresses triple-negative breast cancer tumor growth and brain metastasis by inducing G0/G1 arrest and apoptosis. Cell Death Dis. 2018 Sep 26;9(10):1006. –Fancy RM, Kim H, Napier T, Buchsbaum DJ, Zinn KR, Song Y. Calmodulin antagonist enhances DR5-mediated apoptotic signaling in TRA-8 resistant triple negative breast cancer cells. J Cell Biochem. 2018;119(7):6216–30. –Park S-H, Chung YM, Ma J, Yang Q, Berek JS, Hu MC-T. Pharmacological activation of FOXO3 suppresses triple-negative breast cancer in vitro and in vivo. Oncotarget. 2016 Jul 5;7(27):42110–25.
Valproic acid	Epilepsy	HDAC inhibitor	–Sulaiman A, McGarry S, Lam KM, El-Sahli S, Chambers J, Kaczmarek S, et al. Co-inhibition of mTORC1, HDAC and ESR1α retards the growth of triple-negative breast cancer and suppresses cancer stem cells. Cell Death Dis. 2018 Jul 26;9(8):815. –Tarasenko N, Chekroun-Setti H, Nudelman A, Rephaeli A. Comparison of the anticancer properties of a novel valproic acid prodrug to leading histone deacetylase inhibitors. J Cell Biochem. 2018;119(4):3417–28 –Prestegui-Martel B, Bermúdez-Lugo JA, Chávez-Blanco A, Dueñas-González A, García-Sánchez JR, Pérez-González OA, et al. N-(2-hydroxyphenyl)-2-propylpentanamide, a valproic acid aryl derivative designed in silico with improved anti-proliferative activity in HeLa, rhabdomyosarcoma and breast cancer cells. J Enzyme Inhib Med Chem. 2016;31(sup3):140–9 –Debeb BG, Lacerda L, Larson R, Wolfe AR, Krishnamurthy S, Reuben JM, et al. Histone deacetylase inhibitor-induced cancer stem cells exhibit high pentose phosphate pathway metabolism. Oncotarget. 2016 May 10;7(19):28329–39 –Wiegmans AP, Yap P-Y, Ward A, Lim YC, Khanna KK. Differences in Expression of Key DNA Damage Repair Genes after Epigenetic-Induced BRCAness Dictate Synthetic Lethality with PARP1 Inhibition. Mol Cancer Ther. 2015 Oct;14(10):2321–31
Verapamil	Hypertension, angina pectoris	Calcium channel blocker	–Deshmukh RR, Kim S, Elghoul Y, Dou QP. P-Glycoprotein Inhibition Sensitizes Human Breast Cancer Cells to Proteasome Inhibitors. J Cell Biochem. 2017;118(5):1239–48
Verteporfin	Exudative age-related macular degeneration	Photosensitising agent	–Li Y, Wang S, Wei X, Zhang S, Song Z, Chen X, et al. Role of inhibitor of yes-associated protein 1 in triple-negative breast cancer with taxol-based chemoresistance. Cancer Sci. 2019 Feb;110(2):561–7. –Kim J, Shamul JG, Shah SR, Shin A, Lee BJ, Quinones-Hinojosa A, et al. Verteporfin-Loaded Poly(ethylene glycol)-Poly(beta-amino ester)-Poly(ethylene glycol) Triblock Micelles for Cancer Therapy. Biomacromolecules. 2018 13;19(8):3361–70 –Andrade D, Mehta M, Griffith J, Panneerselvam J, Srivastava A, Kim T-D, et al. YAP1 inhibition radiosensitizes triple negative breast cancer cells by targeting the DNA damage response and cell survival pathways. Oncotarget. 2017 Nov 17;8(58):98495–508
Warfarin	Prophylaxis of systemic embolism, of venous thrombosis and pulmonary embolism.	Vitamin K antagonist	–Beaudin S, Kokabee L, Welsh J. Divergent effects of vitamins K1 and K2 on triple negative breast cancer cells. Oncotarget. 2019 Mar 19;10(23):2292–305
Zoledronic acid	Osteoporosis, prophylaxis of skeletal fractures and treat hypercalcemia of malignancy, treat pain from bone metastases	Bone resorption inhibitor	–Liu H, Wang S-H, Chen S-C, Chen C-Y, Lin T-M. Zoledronic acid blocks the interaction between breast cancer cells and regulatory T-cells. BMC Cancer. 2019 Feb 26;19(1):176. –Cai X-J, Wang Z, Cao J-W, Ni J-J, Xu Y-Y, Yao J, et al. Anti-angiogenic and antitumor effects of metronomic use of novel liposomal zoledronic acid depletes tumor-associated macrophages in triple negative breast cancer. Oncotarget. 2017 Oct 13;8(48):84248–57 –Schech AJ, Kazi AA, Gilani RA, Brodie AH. Zoledronic acid reverses the epithelial-mesenchymal transition and inhibits self-renewal of breast cancer cells through inactivation of NF-κB. Mol Cancer Ther. 2013 Jul;12(7):1356–66 –Ibrahim T, Liverani C, Mercatali L, Sacanna E, Zanoni M, Fabbri F, et al. Cisplatin in combination with zoledronic acid: a synergistic effect in triple-negative breast cancer cell lines. Int J Oncol. 2013 Apr;42(4):1263–70 –Ibrahim T, Mercatali L, Sacanna E, Tesei A, Carloni S, Ulivi P, et al. Inhibition of breast cancer cell proliferation in repeated and non-repeated treatment with zoledronic acid. Cancer Cell Int. 2012 Nov 22;12(1):48. –Gschwantler-Kaulich D, Weingartshofer S, Grunt TW, Mairhofer M, Tan Y, Gamper J, et al. Estradiol impairs the antiproliferative and proapoptotic effect of Zoledronic acid in hormone sensitive breast cancer cells in vitro. PLoS ONE. 2017;12(9):e0185566 –Tripathi R, Singh P, Singh A, Chagtoo M, Khan S, Tiwari S, et al. Zoledronate and Molecular Iodine Cause Synergistic Cell Death in Triple Negative Breast Cancer through Endoplasmic Reticulum Stress. Nutr Cancer. 2016 Jun;68(4):679–88.

**Table 1.** Characteristics of clinical studies about repurposing of old drugs for TNBC treatment.

Reference	Study type	Database used (if observational) and data source type	Country	Study period	Population	Main exclusion criteria	Drugs of interest
**Bayraktar *et al* 2012** [10]	Retrospective study	Breast Cancer Management System (Medical records and pharmacy data)	USA	1995–2007	Women with TNBC who received adjuvant chemotherapy	–Metastatic or bilateral disease –Prior history of cancer –Resolved gestational diabetes –Diabetes diagnosed after adj chemotherapy	Metformin
**Botteri *et al* 2013** [11]	Retrospective cohort study	Breast Cancer and Cardiology Division Databases of the European Institute of Oncology of Milan (Disease registries)	Italy	1997–2008	Postmenopausal women operated for early primary TNBC	History of invasive cancer or metastatic disease	Beta blockers
**Melhem-Bertrand *et al* 2011** [12]	Retrospective cohort study	Breast Cancer Management System Database (Medical chart and pharmacy data)	USA	1995–2007	Women with invasive TNBC treated with neoadjuvant anthracylines and taxane	–BB after neoadjuvant chemotherapy –Unknown receptors expression status –Incomplete records longer than 9 months between neoadjuvant chemotherapy initiation and surgery –Bilateral BC	Beta blockers
**Spera *et al* 2017 (1)** [13]	Retrospective cohort study	Data from a randomised, double blind clinical trial (ROSE/TRIO-012)	Multicentric	–	Women with advanced TNBC	–	Beta blockers
**Spera *et al* 2017 (2)** [13]	Retrospective cohort study	Data from a randomised, double blind clinical trial (BCIRG-005)	Multicentric	–	Women with node positive TNBC	–	Beta blockers
**Chan *et al* 2017** [14]	Phase II, open label, single arm study	–	–	–	Women with stage II/III TNBC	–Patients who have had chemotherapy or radiotherapy within 6 weeks prior to entering the study –Pregnant women	Tetrathio-molybdate
**Hasegawa *et al* 2015** [15]	Phase II, open label, randomised study	–	Multicentric (Japan)	2010–2012	Women with stage IIA/IIIB TNBC	–Bilateral breast cancer or inflammatory breast cancer –Distant metastasis –History of chemotherapy, endocrine therapy, or radiotherapy	Zoledronic acid
**Ishikawa *et al* 2017** [16]	Phase II, open label, randomised study	–	Multicentric (Japan)	2010–2012	Women with stage IIA/IIIB TNBC	–Bilateral breast cancer or inflammatory breast cancer –Distant metastasis –History of chemotherapy, endocrine therapy, or radiotherapy	Zoledronic acid
**Retsky *et al* 2012** [17]	Retrospective study	Data from medical records	Belgium	2003–2008	Women who underwent mastectomy with axillary dissection	–Previous ipsilateral surgery for breast cancer were excluded	Ketorolac
**Chow *et al* 2013** [18]	Phase II, multicentre, open-label, single-arm study (OOTR-N001 study)	–	–	2006–2010	Women with primary breast cancer	–Distant metastasis –Multiple, bilateral breast cancer –Postmenopausal patients with both positive estrogen and progesterone receptor status and negative lymph node involvement –Pregnant women or women with suspected pregnancy –Prior history of invasive breast cancer	Celecoxib
**Shiao *et al* 2017** [19]	Retrospective study	Data from University of texas Southwestern (UTSW) TNBC registry (Medical records)	USA	1998–2016	Women with stageII/III TNBC	–Stage I patients	Aspirin/Clopidogrel
**Williams 2018** [20]	Retrospective study	Electronical medical records	USA	2005–2013	Women with primary operable stages I-III breast cancer	–Not clear use of aspirin –Not surgery –Not primary –Lack of follow up	Aspirin
**Pierga *et al* 2010** [21]	Phase II, randomised study (Remagus 02)	–	–	2004–2000	Women with stageII/III breast cancer	–	Celecoxib
**Tsubamoto *et al* 2014** [22]	Retrospective study	Kohnan hospitals (Medical records)	Japan	2008–2012	Women with TNBC	–Visceral (lungs, brain, and liver) metastasis	Itraconazole
**Wang *et al* 2015** [23]	Phase II, open label, randomised study	–	–	–	Women with metastatic or recurrent breast cancer	–Brain metastases –Prior chemotherapy in the metastatic setting	Esomeprazol
**Nanda *et al* 2016** [24]	Phase I, randomised	–	USA	–	Metastatic or locally advanced breast cancer	–Allergy or hypersensitivuty to mifepristone, paclitaxel –Received more than 4 prior cytotoxic therapies for metastatic disease or prior nab-paclitaxel or mifepistone. –Pregnant or breast feeding	Mifepristone
**Lacerda *et al* 2014** [25]	Retrospective study	IBC database - Breast Cancer Management System at MD Anderson Cancer Center (Medical records)	USA	1995–2011	Patients with Inflammatory breast cancer	–Stage IV patients –Patients who did not receive adj postmastectomy radiotherapy –Patients with locoregional recurrence prior to radiation	Statins
**Shaitelman *et al* 2017** [26]	Retrospective study	Data from MD Anderson Cancer Center (Medical records)	USA	1997–2012	Women with invasive, non-metastatic TNBC	–	Statins

**Table 2.** Outcomes for each clinical study.

Reference	ARM1	ARM2	ARM3	Population size (TNBC)	Average age (years) of TNBC patients	Follow up	Outcome	Outcome size	Effect size measures
Bayraktar *et al* 2012 [10]	Diabetic patients **Metformin + Adj chemo +** Adj chemo: –anthracycline +/- taxane –single-agent taxane –other	Diabetic patients **Adj chemo alone** Adj chemo: –anthracycline +/- taxane –single-agent taxane –other	Not diabeticpatients	1,448 patients –ARM1: 63 –ARM2: 67 –ARM3: 1,318	ARM1: Median 53 ARM2: Median 51 ARM3: Median 58	62 months	1) Distant metastasis free survival 2) Overall survival 3) Recurrence free survival	1) ARM1, ARM2, ARM3 0.73 (0.58–0.83), 0.66 (0.52–0.77), 0.60 (0.57–0.62); p = 0.23 1) ARM2 versus ARM1: 1.63 (95% CI: 0.87–3.06) p = 0.13; ARM3 versus ARM1: 1.62 (95%CI: 0.97–2.71) p = 0.06 2) ARM1, ARM2, ARM3: 0.65 (0.51–0.76), 0.64 (0.5–0.75), 0.54 (0.51–0.56); p = 0.38 2) ARM2 versus ARM1: 1.37 (95% CI: 0.78–2.40) p = 0.27; ARM3 versus ARM1: 1.36 (95% CI: 0.87–2.10) p = 0.17 3) ARM1, ARM2, ARM3: 0.67 (0.52–0.79) 0.69 (0.55–0.79), 0.66 (0.63–0.69); p = 0.58 3) ARM2 versus ARM1: 1.22 (95% CI: 0.66–2.28) p = 0.52; ARM3 versus ARM1: 1.28 (95% CI: 0.79–2.08) p = 0.31	–Five years estimates rates between the three groups –Hazard ratio
Botteri *et al* 2013 [11]	**Beta blockers users**	**Beta blockers non users**	–	800 patients	ARM1: Mean 62 ARM2: Mean 59	ARM1: median 72 months ARM2: median 68 months	1) Breast Cancer-related events 2) Distant metastasis 3) Breast Cancer death	1) 13,6% versus 27.9%; p = 0.015 2) 0.32 (95% CI: 0.12–0.90; p = 0.031) 3) 0.42 (95% CI: 0.18–0.97; p = 0.042)	–Five-year cumulative incidence –Hazard ratio
Melhem-Bertrand *et al* 2011 [12]	**Beta blockers +** neoadj therapy	**Beta blockers non users**	–	1.417 patients –ARM1: 102 –ARM2: 1311	ARM1: Mean 47.5 ARM2: Mean 55	ARM1: Median 55 months ARM2: Median 63 months	1) Recurrence free survival 2) Overall survival	1) 0.30; 95% CI: 0.10–0.87; p = 0.027 2) 0.35; 95% CI: 0.12–1.00; p = 0.05	Hazard ratio
Spera *et al* 2017 1 [13]	**Beta blockers users**	**Beta blockers non users**	–	1144 patients –ARM1: 152 –ARM2: 991	ARM1: Median 60 ARM2: Median 53	Median: 25.1 months	1) Progression free survival 2) Overall survival	1) 0.52; 95%CI: 0.34–0.80; p = 0.002 2) 0.87; 95%CI: 0.58–1.31; p = 0.504	Hazard ratio
Spera *et al* 2017 - 2 [13]	**Beta blockers users**	**Beta blockers non users**	–	35 patients	–	–	1) Relapse free survival 2) Overall survival	1) 0.69; 95%CI: 0.35–1.34; p = 0.269 2) 0.73; 95%CI: 0.35–1.48; p = 0.384	Hazard ratio
Chan *et al* 2017 [14]	Tetramyolibdate	–	–	36 patients	–	Median 6.3 years	Event free survival	Stage II/III patients 90% (95% CI: 78%–100%) Stage IV patients: 69% (95% CI: 49%–96%)	Two-year event free rate
Hasegawa *et al* 2015 [15]	**Zoledronic acid + Neoadj chemotherapy** Chemotherapy: Four cycles of FEC100 every 3 weeks followed by 12 cycles of paclitaxel at 80 mg/m2	**Chemotherapy alone** Chemotherapy: Four cycles of FEC100 every 3 weeks followed by 12 cycles of paclitaxel at 80 mg/m2	–	34 patients	–	–	Pathological complete response rates	ARM1: 6/17(35.3%) CI: 12.6–58.0; ARM2: 2/17(11.8%) CI: 0.0–27.1; p = 0.112	Pathological complete response rates
Ishikawa *et al* 2017 [16]	Zoledronic acid + Neoadj chemotherapy Chemotherapy: Four cycles of FEC100 followed by paclitaxel	**Chemotherapy alone** Chemotherapy: Four cycles of FEC100 followed by paclitaxel	–	34 patients	–	–	Three years disease free survival	ARM1: 94.1%; ARM2: 70.6%; p = 0.077	Percentage
Retsky *et al* 2012 [17]	**Ketorolac + Chemotherapy**	**Chemotherapy alone**	–	Not specified	–	27.3 months	Disease free survival	Far superior disease-free survival in the first few years after surgery (no data shown)	–
Chow *et al* 2013 [18]	**Celecoxib (200mg) + Neoadj chemo: ** Chemotherapy: Four cycles of FEC followed by four cycles of docetaxel	–	–	2 patients	–	–	1) Pathological complete response 2) Near Pathological complete response	1) 0% 2) 50%	Percentage
Shiao *et al* 2017 [19]	Antiplatelet users + Possible chemotherapy	Not antiplatelet users + Possible chemotherapy	–	222 patients –ARM1 65 –ARM2 157	ARM1: Median 55 ARM2: Median 50	ARM1: Median: 41.3 ARM2: Median 40.9	1) Five years Disease free survival 2) Five years Overall survival 3) Five years Distant metastasis rate	1) ARM1: 80.4%; ARM2: 62.3%; 0.503 (0.261–0.970) p = 0.04 2) ARM1: 77.2%; ARM2: 69%;0.652 (0.343–1.239) p = 0.192 3) ARM1: 8.8%; ARM2: 31.9%; 0.310 (0.132–0.729) p = 0.007	–Percentage –Hazard ratio
Williams *et al* 2018 [20]	**Aspirin users + Possible chemotherapy**	**Not aspirin users + Possible chemotherapy**	–	147 patients –ARM1: 33 –ARM2: 114	–	–	1) Overall survival 2) Disease free survival	No specific outcome for TNBC comparing ARM1 versus ARM2	Hazard ratio
Pierga *et al* 2010 [21]	**Celecoxib + Chemotherapy** Chemotherapy: Eight cycles of EC-D	**Chemotherapy** Chemotherapy: Eight cycles of EC-D	–	78 patients –ARM1: 44 –ARM2: 34	–	–	Pathological complete response	29.5% (95% CI: 19.7%–40.9%)	Pathological complete response rates
Tsubamoto *et al* 2014 [22]	**Itraconazole + Chemotherapy ** Chemotherapy : docetaxel, carboplatin, and gemcitabine, vinorelbine, bevacizumab	–	–	13 patients	Median: 45	–	1) Response rates 2) Progression free survival 3) Overall survival	1) 62% (95% CI: 35%–88%) 2) 10.8 months (95% CI: 7.6–15.3 months) 3) 20.4 months (95% CI: 13.1–41.4 months)	Pathological complete response rates
Wang *et al* 2015 [23]	**Esomeprazole low dose (80mg) + chemotherapy** Chemotherapy: Docetaxel followed by cisplatin	**Esomeprazole high dose (100mg) + chemotherapy** Chemotherapy: Docetaxel followed by cisplatin	**Chemotherapy** Docetaxel followed by cisplatin	15 patients –ARM1: 2 –ARM2: 6 –ARM3: 7	–	–	Time to progression	1) 10.7 (ARM1+ARM2) and 5.8 months (ARM3); p = 0.011	Median time
Nanda *et al* 2016 [24]	**Mifepristone (300mg) + Paclitaxel**	**Mifepristone (300mg) + Paclitaxel**	**Placebo**	4 patients –No information on treatments	–	–	Treatment response	Three patients have partial response, and one patient complete response	–
Lacerda et al 2014 [25]	Statins + Postmastectomy radiation	Postmastectomy radiation	–	–ARM1: 16 –ARM2: 86	–	Median: 2.5 years	3 years Risk of locoregional recurrence	No specific outcome for TNBC	–
Shaitelman *et al* 2017 [26]	**Statin users**	**Statin users** (patients with lipid/cholesterol values)	Not statin users	-ARM1: 293 -ARM2: 576	–	Median: 75.1 months	ARM1 versus ARM3 1)Recurrence 2)BCa Death ARM2 versus ARM3 3)Recurrence 4)BCa Death	1) 0.82 (95% CI: 0.57–1.16) 2) 0.70 (95% CI: 0.47–1.03) 3) 0.60 (95% CI: 0.36–1.03) 4) 0.51 (95% CI: 0.28–0.93)	Relative risk

**Table 3.** Ongoing trials found in Clinicaltrials.gov.

Drugs (REDO_DB)	Main indication	Mechanism of action	Clinical trial.gov
Acetylsalicylic acid	Analgesia, swelling, prophylaxis of venous embolism and further heart attacks or strokes	Cyclooxygenase inhibitor	(3)
Atorvastatin	Coronary heart disease, acute coronary syndrome	HMGCR inhibitor	NCT03358017 (Recruitment Status : Recruiting); NCT03872388 (Recruitment Status : Recruiting); NCT02201381 (Recruitment Status : Recruiting)
Celecoxib	OA, RA, JRA, AS, acute pain, primary dysmenorrhea	Cyclooxygenase inhibitor	NCT03599453 (Recruitment Status : Recruiting)
Doxycycline	Respiratory/urinary tract/ophtalmic infection	Metalloproteinase inhibitor	NCT02201381 (Recruitment Status : Recruiting)
Epalrestat	Diabetes	Aldose reductase inhibitor	NCT03244358 (Recruitment Status : Recruiting)
Flucytosine	Candida and/or Cryptococcus	Other antifungal	NCT02576665 (Recruitment Status : Active)
Imipramine	Depression	Norepinephrine reputake inhibitor|serotonin reuptake inhibitor	NCT03122444 (Recruitment Status : Not yet recruiting)
Indomethacin	Analgesia	Cyclooxygenase inhibitor	NCT02950259 (Recruitment Status : Active)
Lansoprazole	Antacid	ATPase inhibitor	NCT03794596 (Recruitment Status : Not yet recruiting)
Leflunomide	Arthritis	Dihydroorotate dehydrogenase inhibitor|PDGFR tyrosine kinase receptor inhibitor	NCT03709446 (Recruitment Status : Recruiting)
Mebendazole	Parasitic infection	Tubulin polymerisation inhibitor	NCT02201381 (Recruitment Status : Recruiting)
Metformin	Diabetes	Insulin sensitizer	NCT01650506 (Recruitment Status : Completed); NCT02201381 (Recruitment Status : Recruiting)
Mifepristone	Abortifacient	Glucocorticoid receptor ntagonist|progesterone receptor antagonist	NCT02788981 (Recruitment Status : Recruiting) NCT02014337 (Recruitment Status : Completed)
Omeprazole	Antacid	ATPase inhibitor	NCT02950259 (Recruitment Status : Active)
Ritonavir	Anti-retroviral	HIV protease inhibitor	NCT01009437 (Recruitment Status : Completed)
Zoledronic acid	Osteoporosis, prophylaxis of skeletal fractures and treat hypercalcemia of malignancy, treat pain from bone metastases	Bone resorption inhibitor	NCT03358017 (Recruitment Status : Recruiting); NCT02595138 (Recruitment Status : Active) NCT02347163 (Recruitment Status : Stopped due to the low accrual rate))
